# Combining Chitosan Nanoparticles and Garlic Essential Oil as Additive Fillers to Produce Pectin-Based Nanocomposite Edible Films

**DOI:** 10.3390/polym15102244

**Published:** 2023-05-09

**Authors:** Vanessa Solfa dos Santos, Marcos Vinicius Lorevice, Graziela Solferini Baccarin, Fabíola Medeiros da Costa, Renan da Silva Fernandes, Fauze A. Aouada, Márcia Regina de Moura

**Affiliations:** 1 Hybrid Composites and Nanocomposites Group (GCNH), Department of Physics and Chemistry, School of Engineering, São Paulo State University (UNESP), Ilha Solteira 15385-000, Brazil; 2 Brazilian Nanotechnology National Laboratory (LNNano), Brazilian Center for Research in Energy and Materials (CNPEM), Campinas 13083-970, Brazil; 3 Chemistry Department, Center for Exact Sciences, Federal University of São Carlos (UFSCar), Rodovia Washington Luís, Km 235, 10 SP 310, São Carlos 13565-905, Brazil

**Keywords:** biopolymers, nanoparticles, garlic essential oil, packaging

## Abstract

Edible films were produced by combining a pectin (PEC) matrix with chitosan nanopar-ticle (CSNP), polysorbate 80 (T80), and garlic essential oil (GEO) as an antimicrobial agent. CSNPs were analyzed for their size and stability, and the films, throughout their contact angle, scanning electron microscopy (SEM), mechanical and thermal properties, water vapor transmission rate, and antimicrobial activity. Four filming-forming suspensions were investigated: PGEO (control); PGEO@T80; PGEO@CSNP; PGEO@T80@CSNP. The compositions are included in the methodology. The average particle size was 317 nm, with the zeta potential reaching +21.4 mV, which indicated colloidal stability. The contact angle of the films exhibited values of 65°, 43°, 78°, and 64°, respec-tively. These values showed films with variations in hydrophilicity. In antimicrobial tests, the films containing GEO showed inhibition only by contact for *S. aureus*. For *E. coli*, the inhibition occurred in films containing CSNP and by direct contact in the culture. The results indicate a promising al-ternative for designing stable antimicrobial nanoparticles for application in novel food packaging. Although, it still shows some deficiencies in the mechanical properties, as demonstrated in the elongation data.

## 1. Introduction

Biopolymers have been called a greener and more sustainable alternative raw material to replace petroleum-based polymers in the production of food packaging (edible films and coatings) due to their low toxicity, biocompatibility, renewability, and biodegradability [1]. From several biopolymers studied in food packaging development, Pectin (PEC) has risen as an eco-friendly option for coating and film applications due to its film-forming ability and greater and greener availability from citrus fruits [2]. PEC is an anionic linear polysaccharide primarily composed of α(1,4) linked D-galacturonic acid chains, being classified according to its degree of esterification: high methoxylation (HMD, when half or more of the carboxyl groups are esterified) and low methoxylation degree (LMD, when less than half of the carboxyl groups are esterified) [3]. These characteristics strongly influence physicochemical and functional properties, such as gelling conditions, solubility, temperature, or even polymer modification, allowing the formation of continuous matrices for the preparation of active packaging [4,5].

Despite these ecopromising properties of PEC, PEC-based films usually do not exhibit higher or comparable physical-chemical attributes than conventional packaging, which has demanded different strategies to enhance film properties. To address these requirements, fillers and nanofillers have been successfully incorporated into the PEC matrix, improving mechanical, barrier, and thermal properties of PEC-based composites and nanocomposites, respectively, in equivalent or superior order, turning these materials into a useful alternative for food applications [2]. Nanostructured fillers, especially polymeric nanoparticles (CS, cellulose nanofibrils, etc.) [6], have been widely inserted in the biopolymer matrices improving substantially and effectively the physicochemical properties, mechanical strength, and water vapor permeability [7], as well as in some cases incorporating complementary properties, such as active properties (antioxidant and antimicrobial) [8].

Active packaging has been the focus of intense study in recent decades, and its application goes far beyond just creating a barrier between the internal and external environment. The interest relies on their capability to perform specific functions in food preservation [9]. These functions are linked to the presence of additives substances that exhibit antimicrobial activities, antioxidants, crosslinkers, or pH regulators. Recently, the incorporation of natural active compounds in food packaging has been highlighted since their consumption has been generally recognized as safe (GRAS) by the Food and Drug Administration (FDA, Silver Spring, MD, USA [10]). Among these natural additives, essential oils (EO) have risen as an effective alternative for having antioxidant, antimicrobial, and anti-inflammatory properties related to the presence of terpenes, flavonoids, and other aromatic substances in their composition, in addition to their aroma and taste properties [11,12].

Garlic essential oil (GEO) is extracted from Allium sativum, an edible plant widely applied in culinary fields with related pharmacological and antimicrobial activities [13]. The GEO antimicrobial activity has been linked to a higher quantity of sulfated substances: Allicin (Diallyl Thiosulfinate) and organosulfur compounds [12,14]. Due to the presence of these active substances, the use of GEO in the development of new active food packaging shows up as an effective option to assist food preservation and/or to replace synthetic additives in the food industry.

Even though CSNP and EO have been widely described as efficient alternatives to improve, respectively, physicochemical and active packaging properties, to date, no work combining these compounds to produce PEC-based films has been reported. Thus, the objective of this work was to produce biodegradable biopolymer-based films containing GEO in PEC matrices, investigating the interference of CSNPs and Tween 80 incorporation in terms of mechanical enhancement, hydrophobic and antimicrobial properties provided by GEO into the PEC films for application in edible packaging. The innovation of this work relies on incorporating an active compound and a nanostructured phase to add and enhance antimicrobial and mechanical properties, respectively, for food packaging applications that demand functional properties and resistant film.

## 2. Materials and Methods

Reagents used in the procedures described here were used without further purifi-cation. Chitosan (MW = 71.3 kDa, degree of deacetylation 94%) was bought from Poly-mar^®^ (Fortaleza, Brazil), while methacrylic acid (MAA, MW = 86.1 kDa—CAS-79-41-4) was purchased from Aldrich (St. Louis, MO, USA). Potassium persulphate PA ACS (270.3 g/mol) and Tween 80 (Polysorbate 80, 604.8 g/mol—CAS-9005-65-6) were bought from Dinamica Química Conteporânea Ltd.a^®^ (São Paulo, Brazil) Garlic essential oil (Allium sp.; density of 1.02 g∙ml^−1^; refraction index 1.57) was gently donated for Lapiendrius Flavours (Itaquaquecetuba, Brazil). The high methoxylated pectin (HMP; MD over 50%) was purchased from CP Kelco© (Limeira, Itaquaquecetuba, Brazil).

### 2.1. Preparation and Characterization of Chitosan Nanoparticles in Solution

The CSNPs were synthesized by polymerization of methacrylic acid (MAA) in CS solution according to work first reported by Moura et al. [15]. This synthesis occurs in two subsequent steps: (1) CS was dispersed 0.2% (*w*/*v*) in acidic solution under (methacrylic acid 1% wt.) at constant stirring for 5 h; (2) the solution was inserted into a reflux condenser and potassium persulfate (K2S2O8) was added as an initiator to the CS solution. The system was kept at 70 °C (1 h) to promote the polymerization of methacrylic acid. Further, the suspension was immersed in an ice bath to stop polymerization. The changes in the transparency of the suspension to opalescent dispersion were indicative of CSNP formation [15].

### 2.2. Chitosan Nanoparticle’s Characterization: Hydrodynamic Size Distribution, Polydisperse Index, and Zeta Potential

The mean size distribution, polydisperse index, and zeta potential of CSNPs were obtained through dynamic light scattering in Zetasizer Nano ZS (Malvern Instruments Inc., Irvine, CA, USA). Measurements were performed in triplicate by diluting the CSNP suspension (1:10) in water to avoid a high light scattering rate.

### 2.3. Film Formulations, Preparation, and Characterization

The film-forming composition (FFC) was based on the presence of GEO, CSNPs, and Tween 80 (T80). The components of the fourth different composition are fully described in Table 1 within their correlated symbols. For all the film-forming suspension (FFS), the total weight was kept constant and equal to 100 g. For compositions without CSNPs (PGEO and PGEO@T80), powdered PEC was gently added to water with continuous mechanical stirring (~1500 rpm). GEO and/or T80 were further added, and the suspension was stirred for 12 h. Film-forming suspension comprising CSNP, firstly, PEC power was added to CSNP dispersion, followed by GEO and/or T80 incorporation. Films were prepared through casting-technique: around 40 g of FFS was spread over a polyester substrate (20 × 30 cm, Mylar^®^ polyester, Dupont, Wilmington, DE, USA), in which wet-thickness was controlled through doctor blade-B and left to dry for 48 h at 25 °C.

### 2.4. Water Wettability

The film water wettability was investigated through a contact angle (WCA) of a water droplet (5.0 μL) over the film surface via KSV Instruments (Helsinki, Finland). A total of 60 images were recorded within 60 s. Measurements were taken at 5 random points on the film surface, according to ASTM D5725-99. The WCA mean values were calculated at the right and left ends of the drop.

### 2.5. Scanning Electron Microscopy (SEM)

The cross-section morphology of the cryogenic fracture of the films was obtained via Zeiss Scanning Electron Microscope (EVO LS15), Jena, Germany, operating at a voltage of 5.00 39 kV to 10.00 kV using a thin layer of gold deposited on the samples.

### 2.6. Fourier Transformed Infrared Spectroscopy (FT-IR)

The FT-IR spectra were recorded in a spectrophotometer (Nicolet—NEXUS 670 FT-IR), Norwood, MA, USA operating in 128 scans, frequency range of 400–4000 cm^−1^ and 2 cm^−1^ of resolution. Films were macerated and pressed into KBr pastilles.

### 2.7. Mechanical Properties: Tensile Strength, Strain, and Young’s Modulus

Firstly, the film thickness was determined at five random positions along the sample using digital micrometer No. 7326 (Mitutoyo Corp., Kanagawa, Japan). The thickness means were further used in mechanical and barrier analysis.

The mechanical attributes of Tensile Strength (TS), Strain (%), and Young’s modulus (E) were calculated from a uniaxial test according to ASTM method D882-12 [16] through an Instron Universal Testing Model 3369 (Instron Corp., Norwood, MA, USA). At least ten samples of each film with dimensions of 100 mm length × 13 mm width were cut and analyzed. The films were equilibrated at 33 ± 2% relative humidity (RH) for 48 h before the test.

### 2.8. Water Vapor Transmission Rate

The water vapor transmission rate (WVRT) and film’s permeance were evaluated according to the ASTM E96-80 [17] with some modifications. Briefly, the film samples were cut into a circular format and placed on poly(methyl methacrylate) cups, which were filled with deionized water, so the films acted as a semipermeable barrier between a high- and a low-RH environment. To determine the RH at films’ undersides (%), WVTR (g h^−1^ m^−2^), and permeance (g kPa^−1^ h^−1^ m^−2^), the test cells had their weights measured every two hours for 24 h at 25 ± 1 °C.

### 2.9. Thermal Property: Thermogravimetric Analysis

The thermal degradation analysis was performed in a Thermogravimetric Analyzer TGA-Q500 (TA Instruments, New Castle, DE, USA). The measurements were recorded in the temperature range of 20 to 800 °C (heating rate of 10 °C/min). The nitrogen flow was maintained at 100 mL/min.

### 2.10. Antimicrobial Properties

Antimicrobial activity was evaluated based on disk inhibition tests as fully described by Otoni et al. [18]. Briefly. The disk inhibition test was performed as follows: colonies isolated from the cultures of *E. coli* (ATCC 25922) and *S. aureus* (ATCC 25923) were inoculated into 0.85% (*w*/*v*) NaCl solution until 0.5 McFarland standard turbidity matched (108 CFU mL^−1^). The suspensions were spread out over solidified Mueller Hinton agar. Discs with diameters of 0.5, 0.6, 0.7, and 0.8 cm were exposed to UV light (110 V and 254 nm) for 2 min on each side before being placed in contact with the bacterial surface, and the plates were incubated at 37 °C for 24 h in the appropriate incubation chamber. The inhibition zones (diameters around the films) were measured to the nearest 0.01 mm with a digital caliper (Mitutoyo Corp., Kanagawa, Japan). The inefficiency in inhibiting microbial growth was considered when inhibition zones were not detected, and the area was assigned as zero.

## 3. Results

### 3.1. CNPS Characterization

The particle size distribution is an important characteristic of nanoparticles since the size is an essential factor that drives their good performance when incorporated into a polysaccharide-based matrix. The mean size obtained for CSNPs was 317 ± 2 nm, in accordance with previous works on CSNPs [19,20,21]. Aranda-Barradas et al. [22] reported regardless of the other factors that can affect the diameter of the particles (CS content or MMA precursor, for instance), chitosan-based systems prepared through via in situ polymerization or the precipitation of preformed polymers usually provide CSNP size diameters ranging from 50 to 300 nm. According to Moura et al. [15], the CSNP is usually spherical and stable. This stability can be measured through zeta potential. Here, CSNPs exhibited a zeta potential of 21.4 ± 0.9 mV, indicating a stable suspension since the values are between the modulus, 20 to 40 mV. In the case of CSNPs, this increase is related to the amount of chitosan; consequently, there will be an increase in the concentration of NH_3_^+^ ions, resulting in a greater surface positive charge [23].

### 3.2. Pectin/GEO Films Characterization

#### 3.2.1. Visual Appearance

**Figure 1:**
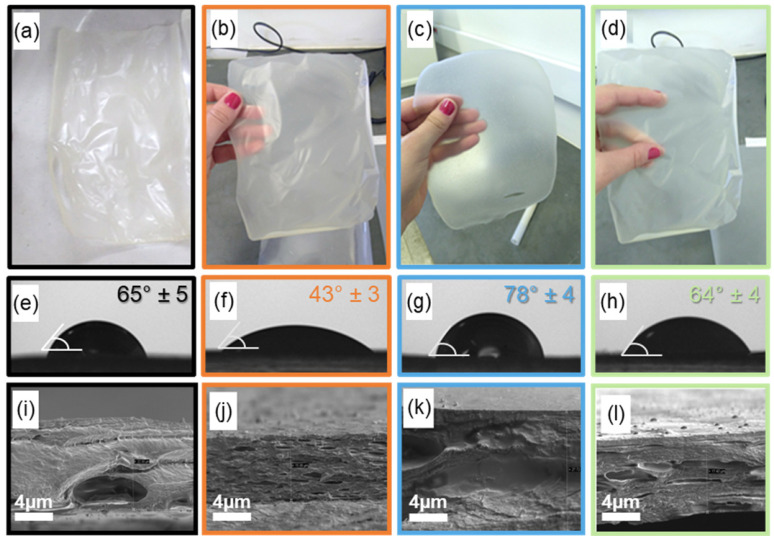
Visual **(a**–**d**), superficial (**e**–**h**), and internal SEM (**i**–**l**) characteristics of pectin-based: PGEO(**a**,**e**,**i**); PGEO@T80 (**b**,**f**,**j**); PGEO@CSNP (**c**,**g**,**k**); PGEO@T80@CSNP (**d**,**h**,**l**).

Figure 1 summarizes these data on visual, superficial, and internal film structure. From Figure 1a–d, differences in the film appearance can be noted when the first Tween 80 and/or CSNPs were incorporated. PGEO@T80 films seemed more flexible than PGEO, indicating that the surfactant could be plasticizing the PEC matrix or even facilitating some compatibilization of GEO hydrophobic components with the polysaccharide matrix. Interestingly, CSNPs made the film extremely homogeneous and apparently more flexible than PGEO. In addition, reduced transparency may be linked to a less crystalline portion of PEC when the nanofiller is added. This behavior was not observed when Tween 80 was added to the film composition. However, despite looking more brittle than PGEO@CSNP, PGEO@T80, and PGEO@T80@CSNP look similar, without fissures or phase separation, revealing the good compatibilization of these three components (GEO, T80, and CSNP) within the polymeric matrix.

#### 3.2.2. Water Wettability

The water contact angle (WCA) measurement gives the wettability (hydrophilic/hydrophobic nature) of the surface of the films. Values greater than 90° indicate surfaces intrinsically hydrophobic, while values below 90° suggest more hydrophilic surfaces [24]. The results can be seen in Figure 1e–h. All films have hydrophilic surfaces, with the PGEO film (Figure 1e) showing a WCA of 65°. By adding surfactant Tween 80 (Figure 1f), the WCA decreased to 43°, indicating that this compound possibly provided greater affinity between its surface and the liquid, turning it more hydrophilic. This may be correlated to a possible plasticizing effect of the surfactant in the matrix, where the hydrophilic part of the surfactant interacts with water, facilitating its presence between the polymer chains [25]. In this context, there is a decrease in the polymer-polymer interactions and the migration of the excess to the surface, increasing the hydrophilicity of the film [22]. Equally, the film containing CSNPs (Figure 1g) exhibited WCA equal to 78°, but when Tween 80 (Figure 1h) was added, the WCA reached 64°. CSNPs may provide the film with a denser matrix caused by favoring the aggregation of hydrophobic agents present in the essential oil during the drying process [26,27]. In general, although all compositions contain GEO, the compositions incorporated with Tween 80 showed low hydrophobicity, considering that the surfactant mediates the oil/water interface, hindering the hydrophobic effect in the mediations of the film surface. Given the results, it is possible to affirm that the chemical composition of the material directly influences the hydrophobic characteristics of the final film.

#### 3.2.3. Internal Morphology: SEM Analysis

The cryogenic fracture micrograph of the films is intended to investigate the effect of the different nanofiller and surfactants in the films containing GEO. Figure 1i–l shows the cross-sections of the PEC-based films. The control film GEO (Figure 1i) presented a compact and ordered structure, suggesting miscibility among some components of the EO and PEC matrix. In films containing Tween 80 (Figure 1j), internal pores with variable dimensions distributed randomly within the matrix can be observed, which could reduce hydrophobic aspects and increase flexibility, providing the film with a more amorphous structure [28,29]. When CSNPs were incorporated into the film composition (Figure 1k), the polymeric matrix was denser and more compact than the e size indicate that CSNPs acted as reinforcing agents. control film (Figure 1i). The uniformity of its internal structure and the decrease in pore size indicate that CSNPs acted as reinforcing agents.

#### 3.2.4. FT-IR Analysis

**Figure 2:**
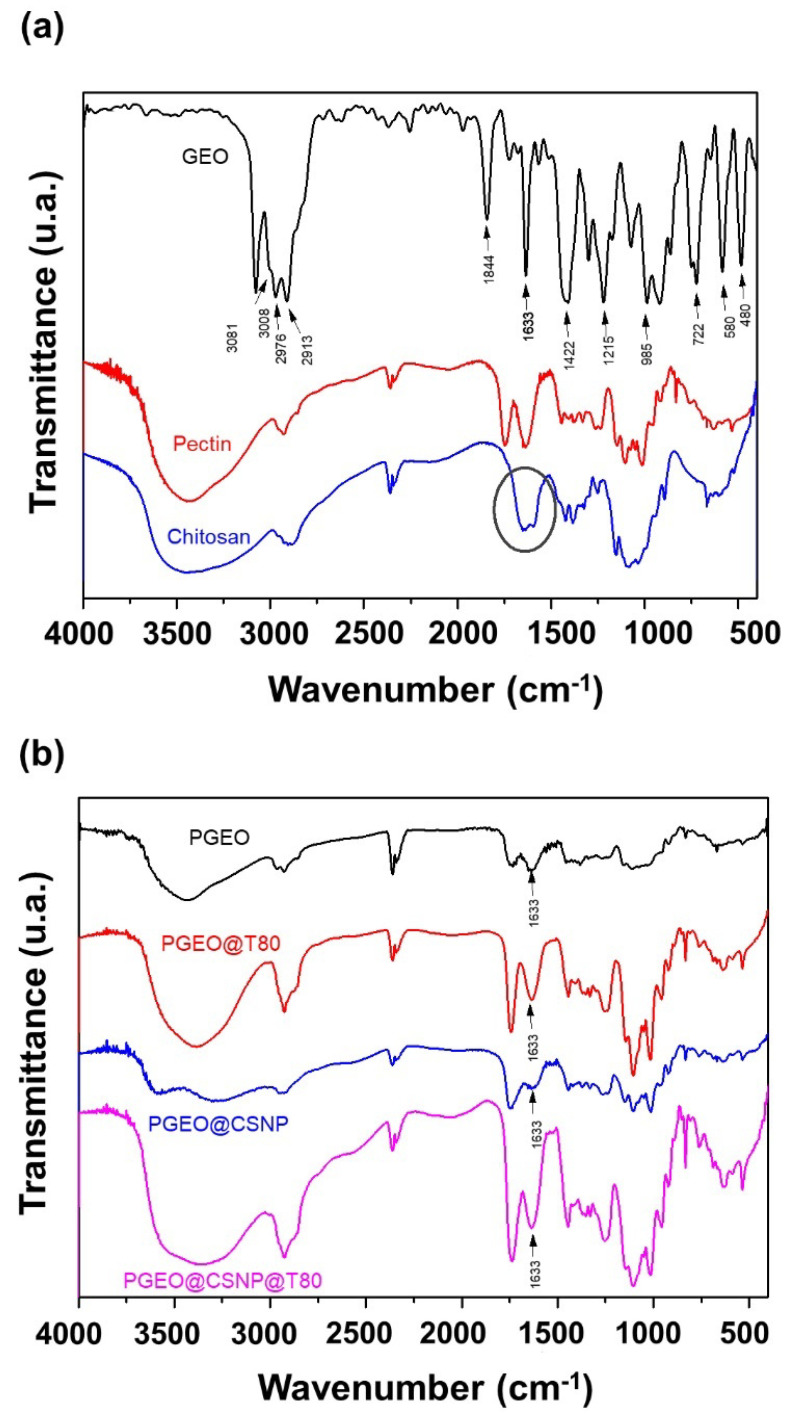
Infrared spectra of (**a**) films compositions: garlic essential oil (GEO, black), pectin (P, red, and chitosan (CS, blue); (**b**) pectin-based films: PGEO (black); PGEO@T80 (red); PGEO@CSNP (blue); PGEO@T80@CSNP (pink).

Figure 2 shows the infrared spectra of the different film compositions and their components. The spectrum of the GEO (Figure 2a) shows the typical four bands in the region between 3100 and 2900 cm^−1^. The first, at 3081 cm^−1^, corresponds to the asymmetric stretching vibration of CH_2_, and the second (3011–3007 cm^−1^) relates to the C-H stretching. The third, from 2980 to 2978 cm^−1^, shows the symmetric stretching vibration of CH_2_, and the fourth, between 2914 and 2912 cm^−1^, the stretching of—CH2-. The bands 1428 and 1401 cm^−1^ are attributed to the stretching of -CH_2_-, and at length 1217 cm^−1^ concerns the stretching CH_2_=CH-, which are associated with the presence of GEO in the films [8,13,30].

Figure 2a,b show the infrared spectra of the neat CS and CSNPs, respectively. The band between 1657 cm^−1^ and 1598 cm^−1^ (Figure 2a, black circle) refers to the amine group of the CS structure, which is shifted (1638 and 1545 cm^−1^) due to the interaction among the amine group of CS (-NH_3_^+^) and the carboxylic groups (COO−) from PMMA. The broad band at 3419 and 2859 cm^−1^ assigned to asymmetric/symmetric stretching of the primary amines (NH_2_) and O–H stretching (Figure 2b) from intermolecular/intramolecular hydrogen bonds) [13,30]. Moreover, peaks at 1750 cm^−1^ and 1640 cm^−1^ are assigned to the stretching of carboxylate ions COO−. In addition, the weaker symmetric COO− stretching is followed by moderately intense absorption patterns between 1300 and 800 cm^−1^, a PEC-fingerprint region, which is also visualized in the spectra of the films (Figure 2b).

In terms of PGEO-filler interactions (T80 and CSNP), a way of identifying is by investigating shifts in characteristic bands of each compound. Here an increase in intensity from 3600 to 3000 cm^−1^ when Tween 80 was added to PGEO films was noted, which can be linked to the OH- groups from surfactant molecules. The typical GEO bands between 3100–2920 cm^−1^ were lower in PGEO@CSNP than in PGEO. Interestingly, CSNP incorporation did not improve this band signal but increased the peak around 1630 cm^−1^, those related to amino groups from the CSNP surface. As Tween 80 and CSNPs were combined in PGEO films, both signals can be noted in the coexistence of surfactant molecules (or micelles) and CSNPs through the polymeric matrix.

#### 3.2.5. Film Thickness and Mechanical Properties

**Figure 3:**
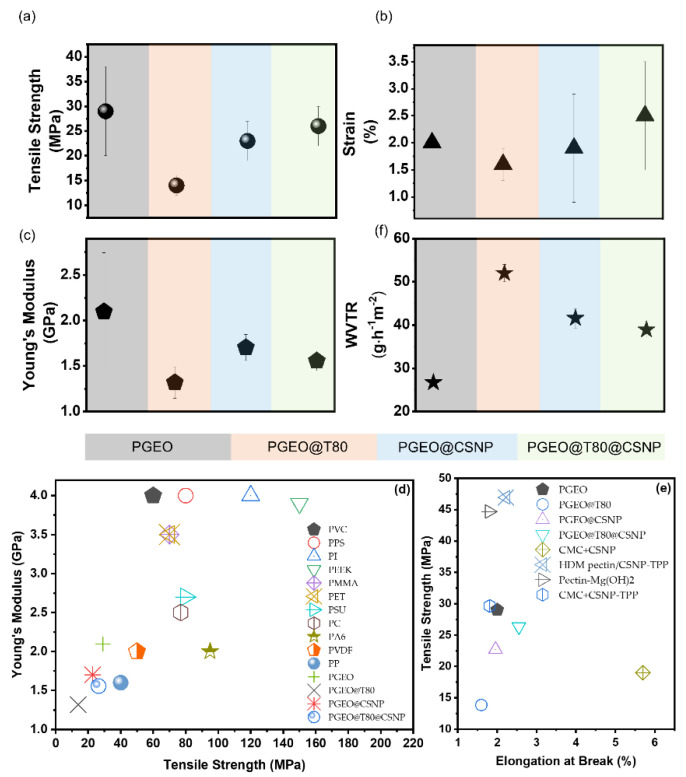
Mechanical properties (tensile strength (**a**), Young’s modulus (**b**), and elongation at break (**c**)) for different film formulations. Young’s modulus vs. Tensile strength for petroleum-based polymeric films (**d**) and Tensile strength vs. elongation at the break (**e**) of biopolymers reinforced with nanofillers [18,42,43,44]. Water vapor transmission rate (WVTR) (**f**) of all the film-forming compositions.

The film thickness showed statistical differences (*p* < 0.05) when CSNPs were incorporated, showing dependency on film content instead of the preparation method or drying conditions. While PGEO and PGEO@T80 film thickness remained unaffected at around 21 ± 5 µm for both compositions, the films containing CSNPs were thicker than those without nanofiller: 57 ± 5 µm (PGEO@CSNP) and 31 ± 5 µm (PGEO@T80@CSNP), which suggests that the solid content (CSNPs represent 10% more) interfered in film thickness and Tween 80 made the polymeric matrix more compact and dense even when the solid content increased. The mechanical properties are one of the most important properties of composite films, which can be evaluated in terms of tensile strength (TS), Young’s modulus (YM), and elongation at break (EB). TS represents the film’s resistance when submitted to tensile forces, and the modulus of elasticity provides quantitative values of film stiffness [31,32]. In this context, the addition of CSNPs and/or Tween 80 in the PEC matrix with garlic essential oil was investigated as to how it could impact the mechanical performance of the PEC-based films. Figure 3a–c show the results obtained from the mechanical properties of different film formulations. The TS, EB, and YM ranged from 13.84 to 29.03 MPa, 2.55 to 1.6%, and 1.32 to 2.1 GPa, respectively. The values of TS and YM of the control film (PGEO) had a significant positive difference (*p* < 0.05) in relation to the other compositions, with variations of up to 47.67% and 62.9%, respectively. The PGEO composite was the stiffness film. On the other hand, the EB has no difference based on the film composition. Although the TS and YM of PGEO@T80 and PGEO@T80@CSNP are not higher than the control film (PGEO), they show some similarities with PP or PVDF, as illustrated in Figure 3d. In terms of flexibility (Figure 3e), the nanocomposites obtained here are comparable with the previous works combining PEC and CSNPs.

The main drawback of PEC-based films in applications relies on their limited me-chanical properties, as they become, under specific drying conditions, more rigid and brittle and have a low elongation at break (EB < 25%) [33,34,35,36]. Thus, the improvement of such properties is essential to improve their applicability. It has been reported that several phenolic compounds, including essential oils extracted from plants, are used to improve the functional properties of biopolymers [9,37,38,39]. In addition, they also describe antioxidant activity in these films as an extra property to extend the shelf life of foods, reduce food waste and limit the application of synthetic chemicals, thus meeting the goals of sustainable industrial and social development [40].

Although tensile strength does not vary statistically, the material shows a flexibility improvement tendency. By adding CSNPs, it is possible to observe an increase in elongation in relation to the control film (PGEO). In addition, the insertion of CSNPs into the PGEO@T80 film increased the TS, YM, and EB. Yeddes et al. [41] described the TS values of films with different proportions of chitosan and PEC, in which composite films had a threefold increase when the proportion of chitosan was increased from 3 to 5 parts. This improvement was attributed to the excellent compatibilization of pectin and CS driven by the interaction between the NH3^+^ groups of chitosan and the COO− groups of pectin [41]. Kurek et al. [32] concluded in their study that chitosan flexibility may be caused by the presence of β-(1–4)-D-glucosamine bonds that are absent in PEC polymer chains. Here the lowest EB values may be linked to high interactions among CSNP surface groups (NH_3_^+^) and PEC monomers (-COOH), as indicated by FT-IR analysis (Figure 2). In this case, as already reported by Lorevice et al. [8], CSNPs were distributed evenly among the adjacent polymer chains, strengthening the polymer matrix by intermolecular interactions. This strong interaction reduced the film’s elongation and increased its mechanical strength as it anchored the flow of PEC chains. As a result, these films are attractive for packaging applications that require good mechanical resistance properties (Figure 3).

The main drawback of pectin-based films in applications relies on their limited mechanical properties, as they become, under specific drying conditions, more rigid and brittle and have a low elongation at break (EB < 25%) [33,34,35,36]. Thus, the improvement in such properties is essential to improve their applicability. It has been reported that several phenolic compounds, including essential oils extracted from plants, are used to improve the functional properties of biopolymers [9,37,38,39]. In addition, they also describe antioxidant activity in these films as an extra property to extend the shelf life of foods, reduce food waste and limit the application of synthetic chemicals, thus meeting the goals of sustainable industrial and social development [40].

Although it does not vary statistically, the material shows an improvement ten-dency. By adding CSNPs, it is possible to observe an increase in elongation, which can be related to the composition PGEO@T80@CSNP in relation to the control film (PGEO). In addition, the insertion of CSNPs to the PGEO@T80 film increased the TS, YM, and EB. Yeddes et al. [41] described the tensile strength values of films with different proportions of chitosan and pectin. However, the tensile strength of composite films had a threefold increase when the proportion of chitosan was increased from 3 to 5 parts. The high tensile strength values of these films can be attributed to the excellent association between the NH3^+^ groups of chitosan and the COO− groups of pectin [41]. Kurek et al. [32] concluded in their studies that chitosan flexibility may be caused by the presence of β-(1–4)-D-glucosamine bonds that are absent in pectin polymer chains.

Norcino et al. [45] showed that the addition of copaiba oil nanoemulsions to PEC films led to a considerable increase in EB, with pure PEC films showing elongation of around 1%, with a concomitant decrease in TS. The same behavior was observed in this study (Figure 3a,b). The authors [45] suggested that the plasticizing action of copaiba oil could lead to a decline in intermolecular interactions between the PEC chains and increase the free volume and mobility of the chain, which would eventually form a film with lower strength and greater flexibility. They also attributed the lower TS of the films to the formation of a heterogeneous and incoherent structure in the films caused by the copaiba oil phase.

The addition of the surfactant caused a significant reduction (*p* < 0.05) in the TS when added only to the control film (PGEO). This behavior was also reported by Brandelero, Yamashita, and Grossmann [46]. Cassava starch films with Tween 80 showed a reduction in mechanical strength values due to the plasticizing effect of the surfactant, increasing the free volume between adjacent starch chains and making the structure more flexible. Tween 80 is a hydrophilic surfactant with a higher hydrophilic-lipophilic balance value, and it can interact with water, which weakens the intermolecular hydrogen bond among polysaccharide-functional groups, consequently resulting in decreased mechanical properties [47]. Thus, the nanocomposites obtained in this study exhibited mechanical properties, such as TS, YM, and EB, on par with those reported in studies that used biopolymer matrices and nanoparticles as reinforcing agents.

#### 3.2.6. Water Vapor Transmission Permeability and Permeance

**Figure 4:**
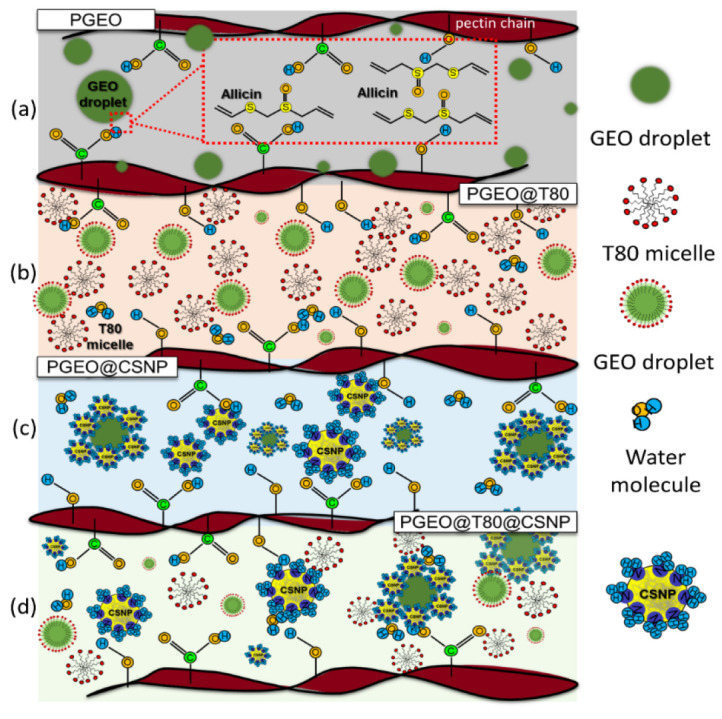
Proposed mechanism of garlic essential oil (GEO), surfactant Tween 80 (T80), and chitosan nanoparticles (CSNP) distribution in pectin matrix. Pectin incorporated with GEO (**a**), GEO@T80 (**b**), GEO@CSNP (**c**), and GEO@T80@CSNP (**d**).

The edible films’ water vapor transmission rate of the nanocomposites shows de-pendency on the film composition (Figure 3f), and GEO and CSNPs-modified films positively improved barrier properties, while Tween 80 caused the opposite to occur. The PGEO film exhibited a WVTR value of 26.70 ± 0.70 g h^−1^ m^−2^ and permeance of 9.59 ± 0.30 g kPa^−1^ h^−1^ m^−2^. Those values suggest GEO’s efficiency, a mixture of hydrophobic compounds, such as allicin, that decreased WVTR and permeance through the film. Those values were below the WVTR values reported for PEC-based films (96.72 ± 9.60 g h^−1^ m^−2^ and 54.77 ± 10.11 g kPa^−1^ h^−1^ m^−2^) [8,48]. This result may be related to the matrix’s nature and the GEO’s hydrophobicity (Figure 4a). The GEO, dispersed homogeneously through the polymer chains, produces hydrophobic sites that can repulse water molecules and reduce hydrophilic groups to form hydrophilic bonds, thus improving the film’s barrier property [49,50,51,52]. In parallel, high-methoxylated pectin contains fewer carboxyl groups, decreasing the hydrophilic sites available to water to interact and facilitate permeation [50].

In the PGEO films containing Tween 80, the WVTR and permeance values increased to 51.98 ± 1.99 g h^−1^ m^−2^ and 21.42 ± 1.06 g kPa^−1^ h^−1^ m^−2^, respectively. This reduction in the film’s barrier can be linked to the presence of the surfactant molecule [53], as seen in Figure 2j. Tween 80 molecules probably increase the narrow spacing between the PEC chains avoiding chain-chain interaction and allowing greater diffusion of water molecules (Figure 4b) through the matrix. These non-free groups favor greater mobility and, consequently, the permeability of the water molecules. Such interactions can be observed in the representative model in Figure 4c for the films containing Tween 80 [46].

Interestingly, CSNP incorporation had different effects in PGEO and PGEO@T80 nanocomposite films (Figure 3f). For PGEO films, CSNPs promoted WVTR and permeance values up to 16.15 ± 1.06 g h^−1^ m^−2^ and 41.60 ± 2.23 g kPa^−1^ h^−1^ m^−2^, respectively. These values are below the values previously reported [8] but greater than PGEO. On the contrary, CSNP improved the water vapor barrier of PGEO@T80 films: 38.92 ± 1.86 g h^−1^ m^−2^ for WVTR and 14.89 ± 0.86 g kPa^−1^ h^−1^ m^−2^ for permeance, decreasing the effect to Tween 80.

The barrier properties of films depend on factors such as thickness, nanofiller content, porosity, and plasticizer concentration [33,54]. McHugh et al. [17] observed that as film thickness increased, it provided better resistance to mass transfer through it; consequently, the equilibrium water vapor partial pressure at the internal surface of the film increased. It caused an improvement in permeability due to the higher HR gradient between the film and the environment [55,56]. Although PGEO nanocomposites containing CSNPs had thicker films, WVTR and permeance do not depend on the film’s thickness. On the contrary, film composition (CSNP and Tween 80) seems to play the role of a water vapor barrier in the nanocomposites produced in this work.

The CSNPs reduced the effects of surfactant presence. In addition, PGEO@T80, PGEO@CSNP, and PGEO@T80@CSNP demonstrated some reduction in the water vapor barrier. The nanoparticles made the film more compact, as verified with SEM micrographs (Figure 2j–l). Then, the number of pores generated by Tween 80 incorporation was reduced, avoiding empty spaces in the film matrix. Moreover, the presence of nanoparticles distributed throughout the polymeric matrix can promote a tortuous path for water molecules, improving path length for diffusion and, consequently, increasing barrier properties, as illustrated in Figure 4d [42].

#### 3.2.7. Thermogravimetric Analysis

**Figure 5:**
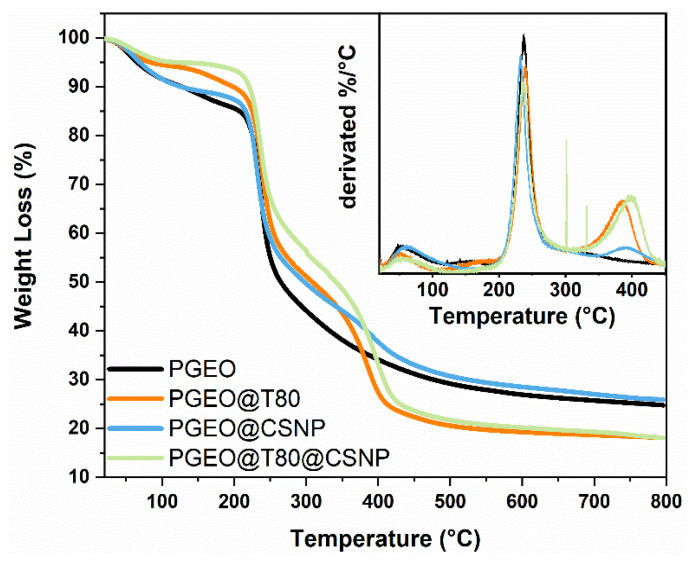
Thermogravimetric scans of composites (PGEO and PGEO@T80) and nanocomposite (PGEO@CSNP and PGEO@T80@CSNP) films.

Thermal analysis is an efficient technique to evaluate the thermal stability of films. The thermograms of the PEC-based films are shown in Figure 5. Generally, the degradation profile of biopolymer-based materials occurs in three stages: dehydration of the material (up to 100 °C) that is naturally retained in the matrix, irreversible degradation of saccharides (~230 °C), and thermal deterioration of organic compounds into CO_2_ and H_2_O above 230 °C [46,50,51,52,53,55,56,57]. Here, a typical initial weight loss stage over 100 °C was observed in all thermogram curves. The presence of T80 added a second weight loss step, probably related to surfactant degradation [50,58]. The degradation onset temperature (T_on_) decreased to 87.73 °C when compared to the thermogram of the PEC matrix. This suggests that the addition of the surfactant decreased the molecular interactions between the adjacent PEC chains made up of hydrogen bonds between the hydroxyl groups of the PEC, as observed in mechanical properties, in which YM and TS reduced after T80 addition (Figure 3a–c). Similar results were mentioned by Norcino et al. [45], who reported a decrease in the Ton of degradation for films containing copaiba oil emulsified with Tween 80 when compared to PEC. Such results were also reported with the addition of extracts in the film composition, as reported in the literature [59,60]. Interestingly, the addition of T80 decreased the water moisture, which was even higher when CSNPs were incorporated into the polysaccharide matrix, which made the nanocomposite matrix more thermally stable. For the films containing CSNPs, a slow mass loss occurs from 140 °C to 200 °C, referring to the decomposition of polymer with low molar mass as NH_3_^+^ [61]. At approximately 243 and 325 °C, the Ton occurs. This event corresponds to the process of dehydration of the anhydrous glucosidic ring present in the chitosan molecule, depolymerization, and side chain decomposition [60,62].

#### 3.2.8. Antimicrobial Properties

**Figure 6:**
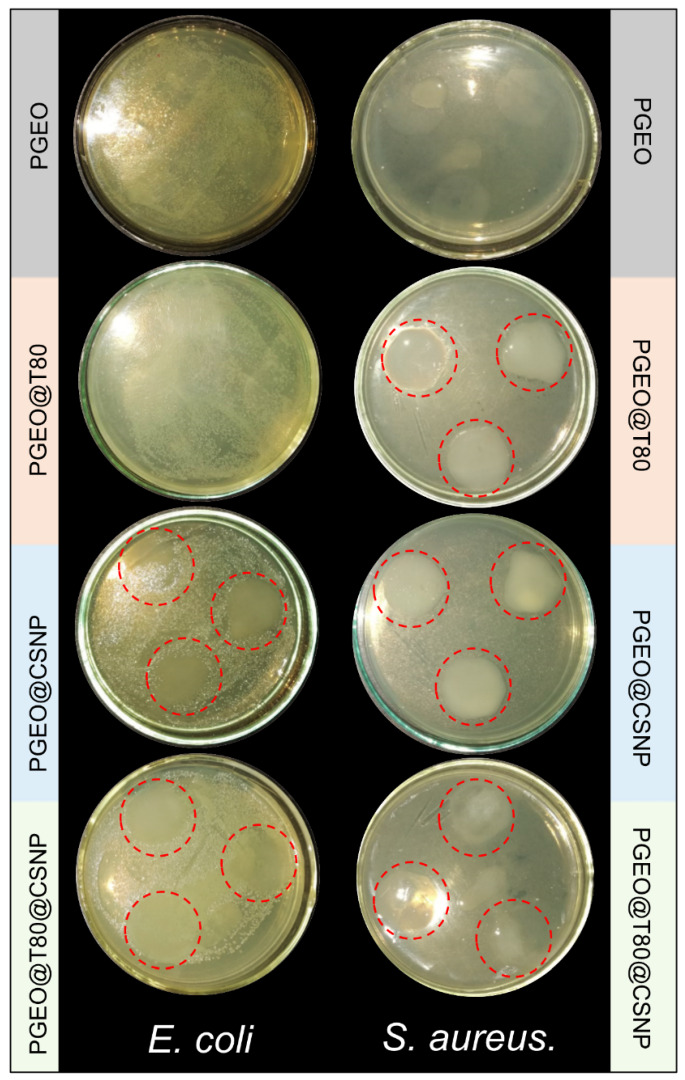
Antimicrobial activity of the films analyzed through an inhibitory halo against *E. coli* and *S. aureus*. The red dashed circles indicate the inhibited grown area.

The antimicrobial activity of the films was analyzed through an inhibitory halo against *E. coli* and *S. aureus* since these microorganisms are widespread pathogens found in food (Figure 6) [33,63]. The inhibition against *E. coli* occurred by contact in the films containing the CSNPs, but not for the control film (PGEO). The results suggest that CS content drives inhibitory activity, and since this polysaccharide is assembled as nanoparticles, the effect may have been intensified. Additionally, the nanostructures have a large surface area. Thus, the ionic interaction of the bacterial cell wall and CSNPs may be enhanced, and consequently, the antimicrobial property. CSNPs may promote cell rupture, changing the membrane permeability, inhibiting the replication of the bacteria DNA, and causing cell death [60,62,64].

GEO and T80 did not show an inhibition effect (Figure 6). It may be related to the GEO volatile compounds evaporating themselves even before diffusion on the Agar, leading to a decrease in the antimicrobial property of the film. The Agar plate method has already been related to greater exposure of these compounds present in GEO (sulfides) to the environment, decreasing the antimicrobial activity of GEO and turning it undetectable [65]. In addition, Gram-negative food spoilage bacteria and foodborne pathogens such as *E. coli* show higher resistance to various types of EOs due to the complexity of their structure, thus explaining the unsatisfactory antimicrobial effect of the films [11,66]. On the contrary, both CSNPs and GEO drove the inhibitory activity of the films for the *S. aureus* bacteria. Previous studies have reported a more synergic effect of CS and EOs against Gram-positive bacteria than Gram-negative bacteria due to the difference in the outer membrane characteristics. These finds explain why all films showed inhibitory activity against *S. aureus*, while for *E. coli*, only those containing CSNPs were effective in inhibition [11,67]. Among several novel packaging types with the potential to reach the final consumers, active packaging is a promising way to slowly release functional additives to food and avoid food spoilage. Moreover, considering the growing concerns about reducing the disposal of plastics in the environment, the suggested applications for biopolymer films based on the results obtained in this work expand the applications already reported for active and edible films. Based on the obtained results, the tested films are not a suitable medium for the microorganisms. Since microorganisms do not grow on the surface of many materials, which does not mean that these materials have antimicrobial activity, the authors believe that additional tests to demonstrate the antimicrobial activity should be performed and presented in future work.

## 4. Conclusions

Herein pectin-based nanocomposite films were produced combining GEO, Tween 80, and CSNPs. The CSNPs synthesized here reached a size distribution of 317 ± 2 nm, and colloidal stability was verified by zeta potential data over 20 mV. GEO presence in the composition of the films caused a decrease in water affinity on the film’s surface, thus increasing their hydrophobicity. The SEM analysis showed Tween 80 producing pores in the film’s structure, which were reduced when CSNPs were incorporated, indicating a reinforcing effect, which was verified by mechanical properties results. Furthermore, GEO addition generated PEC-based films with low WVTR and CSNPs improved the water vapor barrier for nanocomposites containing Tween 80. The probable mechanism is related to the increased barrier property, which was the tortuous path created by CSNPs increasing the path length for water diffusion.

The results from the antimicrobial activity showed that all films containing CSNPs presented inhibition only by contact for Gram-positive bacteria (*S. aureus*) and Gram-negative (*E. coli*). For films containing GEO and CS, the synergistic effect of these fillers contributed to the improvement of inhibitory activity against *S. aureus*. For bacteria of the *E. coli* type, only the films containing nanoparticles were more active. Based on the obtained results, it can only be concluded that the tested films are not a suitable medium for the microorganisms. Though extremely low elongation at break shows that a sample with such composition is not acceptable for application in food packaging, in general, the films presented properties considered satisfactory for application as active edible films, demonstrating homogeneous and continuous appearance, as well as slight transparency and malleability.

## Figures and Tables

**Table 1:** Film-forming composition.

Acronyms	PEC (% wt.)	GEO ^1^ (% *v*/*w*)	CSNP (% wt. PEC)	T80 ^1^ (% wt.)
PGEO	2.0	1.0	-	-
PGEO@T80	2.0	1.0	-	1.0
PGEO@CSNP	2.0	1.0	10	-
PGEO@T80@CSNP	2.0	1.0	10	1.0

^1^ The oil/surfactant weight ratio was equal to 1.

## References

[1] Mollah M.Z.I., Akter N., Quader F.B., Sultana S., Khan R.A. (2016). Biodegradable Colour Polymeric Film (Starch-Chitosan) Development: Characterization for Packaging Materials. Open J. Org. Polym. Mater..

[2] Mellinas C., Ramos M., Jiménez A., Garrigós M.C. (2020). Recent Trends in the Use of Pectin from Agro-Waste Residues as a Natural-Based Biopolymer for Food Packaging Applications. Materials.

[3] Lam M., Shen R., Paulsen P., Corredig M. (2007). Pectin Stabilization of Soy Protein Isolates at Low PH. Food Res. Int..

[4] Kastner H., Einhorn-Stoll U., Senge B. (2012). Structure Formation in Sugar Containing Pectin Gels–Influence of Ca2+ on the Gelation of Low-Methoxylated Pectin at Acidic PH. Food Hydrocoll..

[5] Galus S., Lenart A. (2013). Development and Characterization of Composite Edible Films Based on Sodium Alginate and Pectin. J. Food Eng..

[6] Spricigo P.C., Pilon L., Trento J.P., de Moura M.R., Bonfim K.S., Mitsuyuki M.C., Mattoso L.H.C., Ferreira M.D. (2021). Nano-Chitosan as an Antimicrobial Agent in Preservative Solutions for Cut Flowers. J. Chem. Technol. Biotechnol..

[7] Antoniou J., Liu F., Majeed H., Qi J., Yokoyama W., Zhong F. (2015). Physicochemical and Morphological Properties of Size-Controlled Chitosan–Tripolyphosphate Nanoparticles. Colloids Surf. A Physicochem. Eng. Asp..

[8] Lorevice M.V., Otoni C.G., de Moura M.R., Mattoso L.H.C. (2016). Chitosan Nanoparticles on the Improvement of Thermal, Barrier, and Mechanical Properties of High- and Low-Methyl Pectin Films. Food Hydrocoll..

[9] Vianna T.C., Marinho C.O., Marangoni Júnior L., Ibrahim S.A., Vieira R.P. (2021). Essential Oils as Additives in Active Starch-Based Food Packaging Films: A Review. Int. J. Biol. Macromol..

[10] Azman N.H., Khairul W.M., Sarbon N.M. (2022). A Comprehensive Review on Biocompatible Film Sensor Containing Natural Extract: Active/Intelligent Food Packaging. Food Control.

[11] Burt S. (2004). Essential Oils: Their Antibacterial Properties and Potential Applications in Foods—A Review. Int. J. Food Microbiol..

[12] Nunes J.C., Melo P.T.S., Lorevice M.V., Aouada F.A., de Moura M.R. (2021). Effect of Green Tea Extract on Gelatin-Based Films Incorporated with Lemon Essential Oil. J. Food Sci. Technol..

[13] Mondéjar-López M., Rubio-Moraga A., López-Jimenez A.J., García Martínez J.C., Ahrazem O., Gómez-Gómez L., Niza E. (2022). Chitosan Nanoparticles Loaded with Garlic Essential Oil: A New Alternative to Tebuconazole as Seed Dressing Agent. Carbohydr. Polym..

[14] Ajami M., Vazirijavid R. (2019). Garlic (*Allium Sativum* L.). Nonvitamin Nonmineral Nutr. Suppl..

[15] de Moura M.R., Aouada F.A., Mattoso L.H.C. (2008). Preparation of Chitosan Nanoparticles Using Methacrylic Acid. J. Colloid Interface Sci..

[16] (2012). Standard Test Methods for Tensile Properties of Thin Plastic Sheeting. Annual Book of American Standard Testing Methods.

[17] Mchugh T.H., Avena-Bustillos F.L., Krochta J.M. (1993). Hydrophilic Edible Films: Modified Procedure for Water Vapor Permeability and Explanation of Thickness Effects. J. Food Sci..

[18] Otoni C.G., Avena-Bustillos R.J., Azeredo H.M.C., Lorevice M.V., Moura M.R., Mattoso L.H.C., McHugh T.H. (2017). Recent Advances on Edible Films Based on Fruits and Vegetables—A Review. Compr. Rev. Food Sci. Food Saf..

[19] Koukaras E.N., Papadimitriou S.A., Bikiaris D.N., Froudakis G.E. (2012). Insight on the Formation of Chitosan Nanoparticles through Ionotropic Gelation with Tripolyphosphate. Mol. Pharm..

[20] Shukla S.K., Mishra A.K., Arotiba O.A., Mamba B.B. (2013). Chitosan-Based Nanomaterials: A State-of-the-Art Review. Int. J. Biol. Macromol..

[21] Hosseini S.F., Soleimani M.R., Nikkhah M. (2018). Chitosan/Sodium Tripolyphosphate Nanoparticles as Efficient Vehicles for Antioxidant Peptidic Fraction from Common Kilka. Int. J. Biol. Macromol..

[22] Aranda-Barradas M.E., Trejo-López S.E., Real A.D., Álvarez-Almazán S., Méndez-Albores A., García-Tovar C.G., González-Díaz F.R., Miranda-Castro S.P. (2022). Effect of Molecular Weight of Chitosan on the Physicochemical, Morphological, and Biological Properties of Polyplex Nanoparticles Intended for Gene Delivery. Carbohydr. Polym. Technol. Appl..

[23] Lorevice M.V., De Moura M.R., Mattoso L.H.C. (2014). Nanocompósito de Polpa de Mamão e Nanopartículas de Quitosana Para Aplicação Em Embalagens. Quim. Nova.

[24] Ortega-Toro R., Jiménez A., Talens P., Chiralt A. (2014). Effect of the Incorporation of Surfactants on the Physical Properties of Corn Starch Films. Food Hydrocoll..

[25] Rodríguez M., Osés J., Ziani K., Maté J.I. (2006). Combined Effect of Plasticizers and Surfactants on the Physical Properties of Starch Based Edible Films. Food Res. Int..

[26] Muscat D., Tobin M.J., Guo Q., Adhikari B. (2014). Understanding the Distribution of Natural Wax in Starch–Wax Films Using Synchrotron-Based FTIR (S-FTIR). Carbohydr. Polym..

[27] Basiak E., Debeaufort F., Lenart A. (2016). Effect of Oil Lamination between Plasticized Starch Layers on Film Properties. Food Chem..

[28] Zhong Y., Li Y. (2011). Effects of Surfactants on the Functional and Structural Properties of Kudzu (*Pueraria Lobata*) Starch/Ascorbic Acid Films. Carbohydr. Polym..

[29] Rubilar J.F., Zúñiga R.N., Osorio F., Pedreschi F. (2015). Physical Properties of Emulsion-Based Hydroxypropyl Methylcellulose/Whey Protein Isolate (HPMC/WPI) Edible Films. Carbohydr. Polym..

[30] Handayasari F., Suyatma N.E., Nurjanah S. (2019). Physiochemical and Antibacterial Analysis of Gelatin–Chitosan Edible Film with the Addition of Nitrite and Garlic Essential Oil by Response Surface Methodology. J. Food Process. Preserv..

[31] Wang H., Ding F., Ma L., Zhang Y. (2021). Edible Films from Chitosan-Gelatin: Physical Properties and Food Packaging Application. Food Biosci..

[32] Kurek M., Benbettaieb N., Ščetar M., Chaudy E., Elez-Garofulić I., Repajić M., Klepac D., Valić S., Debeaufort F., Galić K. (2021). Novel Functional Chitosan and Pectin Bio-Based Packaging Films with Encapsulated Opuntia-Ficus Indica Waste. Food Biosci..

[33] Jamróz E., Tkaczewska J., Juszczak L., Zimowska M., Kawecka A., Krzyściak P., Skóra M. (2022). The Influence of Lingonberry Extract on the Properties of Novel, Double-Layered Biopolymer Films Based on Furcellaran, CMC and a Gelatin Hydrolysate. Food Hydrocoll..

[34] Oyeoka H.C., Ewulonu C.M., Nwuzor I.C., Obele C.M., Nwabanne J.T. (2021). Packaging and Degradability Properties of Polyvinyl Alcohol/Gelatin Nanocomposite Films Filled Water Hyacinth Cellulose Nanocrystals. J. Bioresour. Bioprod..

[35] Oyekanmi A.A., Abdul Khalil H.P.S., Rahman A.A., Mistar E.M., Olaiya N.G., Alfatah T., Yahya E.B., Mariana M., Hazwan C.M., Abdullah C.K. (2021). Extracted Supercritical CO2 Cinnamon Oil Functional Properties Enhancement in Cellulose Nanofibre Reinforced Euchema Cottoni Biopolymer Films. J. Mater. Res. Technol..

[36] Regina S., Poerio T., Mazzei R., Sabia C., Iseppi R., Giorno L. (2022). Pectin as a Non-Toxic Crosslinker for Durable and Water-Resistant Biopolymer-Based Membranes with Improved Mechanical and Functional Properties. Eur. Polym. J..

[37] Jahromi M., Niakousari M., Golmakani M.T. (2022). Fabrication and Characterization of Pectin Films Incorporated with Clove Essential Oil Emulsions Stabilized by Modified Sodium Caseinate. Food Packag. Shelf Life.

[38] do Evangelho J.A., da Silva Dannenberg G., Biduski B., el Halal S.L.M., Kringel D.H., Gularte M.A., Fiorentini A.M., da Rosa Zavareze E. (2019). Antibacterial Activity, Optical, Mechanical, and Barrier Properties of Corn Starch Films Containing Orange Essential Oil. Carbohydr. Polym..

[39] Li Z., Jiang X., Huang H., Liu A., Liu H., Abid N., Ming L. (2022). Chitosan/Zein Films Incorporated with Essential Oil Nanoparticles and Nanoemulsions: Similarities and Differences. Int. J. Biol. Macromol..

[40] Luo Q., Hossen M.A., Zeng Y., Dai J., Li S., Qin W., Liu Y. (2022). Gelatin-Based Composite Films and Their Application in Food Packaging: A Review. J. Food Eng..

[41] Yeddes W., Djebali K., Aidi Wannes W., Horchani-Naifer K., Hammami M., Younes I., Saidani Tounsi M. (2020). Gelatin-Chitosan-Pectin Films Incorporated with Rosemary Essential Oil: Optimized Formulation Using Mixture Design and Response Surface Methodology. Int. J. Biol. Macromol..

[42] De Moura M.R., Lorevice M.V., Mattoso L.H.C., Zucolotto V. (2011). Highly Stable, Edible Cellulose Films Incorporating Chitosan Nanoparticles. J. Food Sci..

[43] Moreira F.K.V., De Camargo L.A., Marconcini J.M., Mattoso L.H.C. (2013). Nutraceutically Inspired Pectin-Mg(OH)2 Nanocomposites for Bioactive Packaging Applications. J. Agric. Food Chem..

[44] Santos V.S., Dos Santos V.S., Fernandes R.d.S., Júnior C.R.F., Aouada F.A., Américo-Pinheiro J.H.P., De Moura M.R. (2021). Evaluation and Characterization of Edible Carboxymethylcellulose Biofilm Containing Chitosan Nanoparticles and Turmeric. Rev. Mater..

[45] Norcino L.B., Mendes J.F., Natarelli C.V.L., Manrich A., Oliveira J.E., Mattoso L.H.C. (2020). Pectin Films Loaded with Copaiba Oil Nanoemulsions for Potential Use as Bio-Based Active Packaging. Food Hydrocoll..

[46] Brandelero R.P.H., Yamashita F., Grossmann M.V.E. (2010). The Effect of Surfactant Tween 80 on the Hydrophilicity, Water Vapor Permeation, and the Mechanical Properties of Cassava Starch and Poly(Butylene Adipate-Co-Terephthalate) (PBAT) Blend Films. Carbohydr. Polym..

[47] Song X., Zuo G., Chen F. (2018). Effect of Essential Oil and Surfactant on the Physical and Antimicrobial Properties of Corn and Wheat Starch Films. Int. J. Biol. Macromol..

[48] Otoni C.G., Pontes S.F.O., Medeiros E.A.A. (2014). Edible Films from Methylcellulose and Nanoemulsions of Clove Bud (*Syzygium aromaticum*) and Oregano (*Origanum vulgare*) Essential Oils as Shelf Life Extenders for Sliced Bread. J. Agric. Food Chem..

[49] Bravin B., Peressini D., Sensidoni A. (2004). Influence of Emulsifier Type and Content on Functional Properties of Polysaccharicle Lipid-Basid Edible Films. J. Agric. Food Chem..

[50] Espitia P.J.P., Du W.X., Avena-Bustillos R.d.J., Soares N.d.F.F., McHugh T.H. (2014). Edible Films from Pectin: Physical-Mechanical and Antimicrobial Properties-A Review. Food Hydrocoll..

[51] Cazón P., Velazquez G., Ramírez J.A., Vázquez M. (2017). Polysaccharide-Based Films and Coatings for Food Packaging: A Review. Food Hydrocoll..

[52] Aitboulahsen M., El Galiou O., Laglaoui A., Bakkali M., Hassani Zerrouk M. (2020). Effect of Plasticizer Type and Essential Oils on Mechanical, Physicochemical, and Antimicrobial Characteristics of Gelatin, Starch, and Pectin-Based Films. J. Food Process. Preserv..

[53] Myllärinen P., Partanen R., Seppälä J., Forssell P. (2002). Effect of Glycerol on Behaviour of Amylose and Amylopectin Films. Carbohydr. Polym..

[54] Giancone T., Torrieri E., Pierro P.D., Cavella S., Giosafatto C.V.L., Masi P. (2011). Effect of Surface Density on the Engineering Properties of High Methoxyl Pectin-Based Edible Films. Food Bioproc. Technol..

[55] Park H.J., Chinnan M.S. (1995). Gas and Water Vapor Barrier Properties of Edible Films from Protein and Cellulosic Materials. J. Food Eng..

[56] Chen H. (1995). Functional Properties and Applications of Edible Films Made of Milk Proteins. J. Dairy Sci..

[57] Martelli M.R., Barros T.T., De Moura M.R., Mattoso L.H.C., Assis O.B.G. (2013). Effect of Chitosan Nanoparticles and Pectin Content on Mechanical Properties and Water Vapor Permeability of Banana Puree Films. J. Food Sci..

[58] Monfregola L., Leone M., Vittoria V., Amodeo P., De Luca S. (2011). Chemical Modification of Pectin: Environmental Friendly Process for New Potential Material Development. Polym. Chem..

[59] Cerruti P., Santagata G., Gomez D’Ayala G., Ambrogi V., Carfagna C., Malinconico M., Persico P. (2011). Effect of a Natural Polyphenolic Extract on the Properties of a Biodegradable Starch-Based Polymer. Polym. Degrad. Stab..

[60] Rejinold N.S., Muthunarayanan M., Muthuchelian K., Chennazhi K.P., Nair S.V., Jayakumar R. (2011). Saponin-Loaded Chitosan Nanoparticles and Their Cytotoxicity to Cancer Cell Lines in Vitro. Carbohydr. Polym..

[61] Thandapani G., Supriya Prasad P., Sudha P.N., Sukumaran A. (2017). Size Optimization and in Vitro Biocompatibility Studies of Chitosan Nanoparticles. Int. J. Biol. Macromol..

[62] Feng Y., Xia W. (2011). Preparation, Characterization and Antibacterial Activity of Water-Soluble O-Fumaryl-Chitosan. Carbohydr. Polym..

[63] Moghimi R., Ghaderi L., Rafati H., Aliahmadi A., Mcclements D.J. (2016). Superior Antibacterial Activity of Nanoemulsion of Thymus Daenensis Essential Oil against *E. coli*. Food Chem..

[64] Medina Jaramillo C., Gutiérrez T.J., Goyanes S., Bernal C., Famá L. (2016). Biodegradability and Plasticizing Effect of Yerba Mate Extract on Cassava Starch Edible Films. Carbohydr. Polym..

[65] Ross Z.M., O’Gara E.A., Hill D.J., Sleightholme H.V., Maslin D.J. (2001). Antimicrobial Properties of Garlic Oil against Human Enteric Bacteria: Evaluation of Methodologies and Comparisons with Garlic Oil Sulfides and Garlic Powder. Appl. Environ. Microbiol..

[66] Yuan G., Chen X., Li D. (2016). Chitosan Films and Coatings Containing Essential Oils: The Antioxidant and Antimicrobial Activity, and Application in Food Systems. Food Res. Int..

[67] Pranoto Y., Salokhe V.M., Rakshit S.K. (2005). Physical and Antibacterial Properties of Alginate-Based Edible Film Incorporated with Garlic Oil. Food Res. Int..

